# The Multiple Roles of Autophagy in Neural Function and Diseases

**DOI:** 10.1007/s12264-023-01120-y

**Published:** 2023-10-19

**Authors:** Yan-Yan Li, Zheng-Hong Qin, Rui Sheng

**Affiliations:** Department of Pharmacology and Laboratory of Aging and Nervous Diseases, Jiangsu Key laboratory of Neuropsychiatric Diseases, College of Pharmaceutical Sciences of Soochow University, Suzhou, 215123 China

**Keywords:** Autophagy, Neurodegenerative diseases, Cerebral ischemia, AMPK, mTOR, Beclin 1, *TP53*, Endoplasmic reticulum stress

## Abstract

Autophagy involves the sequestration and delivery of cytoplasmic materials to lysosomes, where proteins, lipids, and organelles are degraded and recycled. According to the way the cytoplasmic components are engulfed, autophagy can be divided into macroautophagy, microautophagy, and chaperone-mediated autophagy. Recently, many studies have found that autophagy plays an important role in neurological diseases, including Alzheimer's disease, Parkinson's disease, Huntington's disease, neuronal excitotoxicity, and cerebral ischemia. Autophagy maintains cell homeostasis in the nervous system via degradation of misfolded proteins, elimination of damaged organelles, and regulation of apoptosis and inflammation. AMPK-mTOR, Beclin 1, *TP53*, endoplasmic reticulum stress, and other signal pathways are involved in the regulation of autophagy and can be used as potential therapeutic targets for neurological diseases. Here, we discuss the role, functions, and signal pathways of autophagy in neurological diseases, which will shed light on the pathogenic mechanisms of neurological diseases and suggest novel targets for therapies.

## Introduction

Autophagy involves the isolation and delivery of cytoplasmic materials to lysosomes, where lipids, proteins, and organelles are degraded and recycled [1]. Typically, autophagy is classified as microautophagy, macroautophagy, and chaperone-mediated autophagy (CMA) [2]. Autophagy also participates in numerous essential cellular activities and affects many intracellular regulatory pathways involved in diverse processes [3], including development, immunity, longevity, organelle turnover, and apoptosis. Since the 1990s, autophagy research has gradually expanded and developed into one of the most important topics in cell biology. The discovery of the autophagy mechanism earned Dr. Osumi the Nobel Prize in Physiology or Medicine. Researchers have developed molecular tools and drugs that target genes and pathways associated with autophagy, which have advanced the field [4–6].

**Fig. 1:**
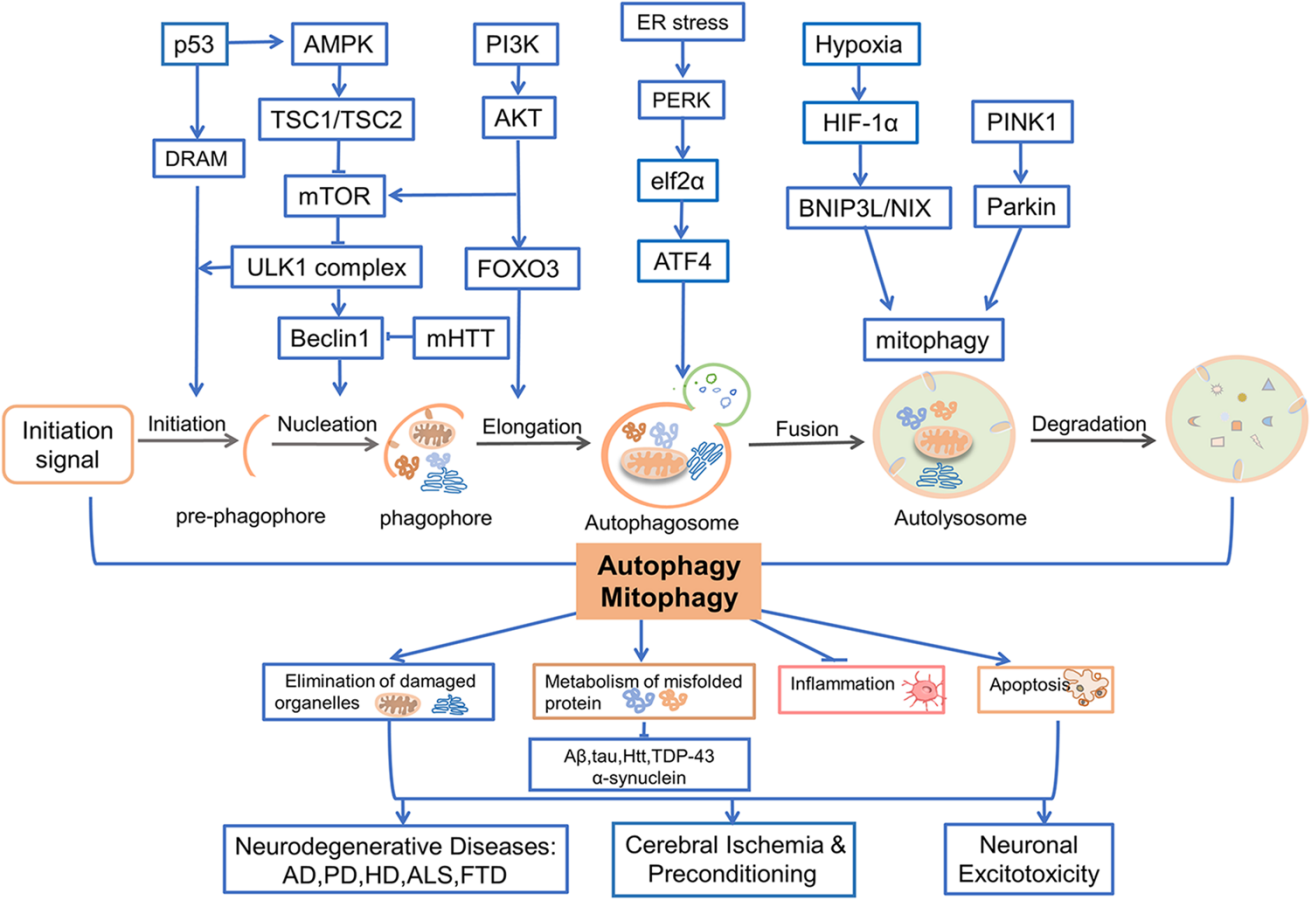
Autophagy in Neurological Diseases. Autophagy plays an important role in the pathogenesis of neurodegenerative disorders, including AD, PD, ALS and FTD, cerebral ischemia, excitotoxicity, and other neurological diseases by eliminating damaged organelles, degrading misfolded proteins, regulating inflammation and apoptosis. The multifunctional nature of autophagy can be attributed to its ability to interact with various key components in the AMPK-mTOR, Beclin 1, TP53, DRAM1, and ER stress signaling pathways. AMPK suppresses mTOR and subsequently triggers the downstream ULK1 complex, which in turn activates Beclin 1, thereby promoting autophagy initiation; activation of this pathway confers protection against AD, PD, and stroke. The AKT/FOXO3 signaling pathway is implicated in cerebral ischemia-induced autophagy. Aβ, tau protein, and α-synuclein hinder autophagy at various stages to promote the progression of AD and PD. The PINK1/parkin pathway facilitates mitophagy for neuroprotection in both diseases. BNIP3L mediates mitophagy in the ischemic brain to prevent injury, while mitophagy also suppresses inflammasome activation to safeguard neurons. AD, Alzheimer’s disease; PD, Parkinson’s disease; HD, Huntington’s disease; ALS, Amyotrophic Lateral Sclerosis; FTD, Frontotemporal Dementia; Aβ, amyloid β-protein; tau, microtubule-associated protein tau; AMPK, Adenosine 5’-monophosphate (AMP)-activated protein kinase; mTOR, mammalian target of rapamycin; ULK1, Unc-51-like kinase; TSC, tuberous sclerosis complex; PI3K, Phosphoinositide-3-kinase; AKT, a serine/threonine kinase; p53, a tumor suppressor protein, and transcription factor; DRAM, DNA damage regulated autophagy modulator; Beclin 1, key regulator of autophagy; mHTT, mutant huntingtin; FOXO3, forkhead box O3; PINK1, PTEN-induced putative kinase 1; Parkin, Parkin RBR E3 ubiquitin-protein ligase; BNIP3L/NIX, Bcl2 interacting protein 3 (BNIP3) and BNIP3-like; ER stress, endoplasmic reticulum stress; PERK, Protein kinase RNA (PKR)-like ER kinase; eIF2α, eukaryotic initiation factor-2α; ATF4, Activating transcription factor 4; HIF-1α, Hypoxia-inducible factor 1

In recent years, autophagy has developed into an important study area, especially in nervous system diseases. Neurons in the nervous system are susceptible to a variety of internal and external injuries, including ischemia/reperfusion (I/R), neurodegeneration, inflammation, energy crisis, metabolic disorders, neurotoxicity *et al.* [7]. Under these stress conditions, autophagy can be activated to varying degrees. Moderate autophagy can maintain neuronal homeostasis and clear protein aggregates or damaged organelles; autophagy also maintains energy balance by recycling the fatty acids, amino acids, and nucleic acids [8]. Therefore, mild to moderate autophagy is a survival mechanism of neurons and can maintain the homeostasis of the central nervous system (CNS). However, continuous or excessive autophagy may contribute to increased cytoplasmic accumulation of autophagosomes and degradation of essential components [9], and even result in autophagic cell death or the implementation of other cell death mechanisms [10, 11]. Here, we review the effect of autophagy in regulating cell metabolism and cell survival in CNS diseases, including neuronal excitotoxicity, neurodegenerative diseases, cerebral ischemia, and ischemic preconditioning. Especially, autophagy is involved in excitotoxicity, degradation of misfolded proteins, and apoptosis in neurological diseases (Fig. 1). These studies shed light on some of the pathogenic mechanisms of neurological diseases and proposed novel therapeutic targets.

## Effects of Autophagy in Neurological Disorders

### Autophagy in Neurodegenerative Diseases

Neurodegenerative disease is a progressive age-related disorder. Since neurons are post-mitotic cells with age-related decline in autophagy, misfolded proteins, and damaged organelles tend to accumulate in neurons, and neurodegenerative diseases are characterized by the accumulation of misfolded proteins [12, 13]. Autophagy is thus considered a cytoprotective mechanism in a variety of neurodegenerative diseases that can mitigate the onset and progression of neurodegenerative lesions [14, 15]. In 2003, Rubinsztein’s lab proved that autophagy and proteasomal pathways are associated with the degradation of α-synuclein in PD. Later, Rubinsztein’s lab [16] and our lab showed that autophagy participates in the degradation of the mutant Huntington protein (aggregated HTT) in HD. Rapamycin, which suppresses mTOR to trigger autophagy, increased the degradation of HTT and improved the neurodegenerative symptoms in mouse and drosophila chorea models [17]. Later studies manifested that activation of autophagy exerts beneficial effects in almost all neurodegenerative diseases, primarily through the removal of cytoplasmic aggregates, such as the α-synuclein in PD, amyloid-β (Aβ) [18] and Tau protein [19] in AD, and the TAR DNA binding protein 43 (TDP-43) aggregates in amyotrophic lateral sclerosis (ALS) [20], mutations in autosomal dominant lateral temporal epilepsy (ADLTE) and Machado-Joseph disease (MJD) [21]or ataxin-3 mutants accumulated in spinocerebellar ataxia type 3 (SCA3) [22, 23].

When the autophagy-associated proteins ATG5 (autophagy-related 5) or ATG7 were specifically knocked out in mouse neurons, basal autophagy level in the brain was markedly decreased, while ubiquitinated proteins, p62, and non-degradable protein aggregates accumulated [24]. Many neurons were lost in the brain and cerebellar cortex of *Atg7* deficient mice [25, 26]. In the absence of *Atg5*, mice developed progressive motor dysfunction [27]. The above studies emphasize the importance of autophagy in preventing neurodegeneration. Similarly, during aging or AD, neuronal autophagy activity in the hippocampus or ventral tegmental area (VTA) decreases and Aβ accumulates. Restoring autophagy effectively reduced Aβ levels and reversed neuronal degeneration and memory deficits [28, 29]. Interestingly, early enhancement of autophagy produces an initial neuroprotective response to cellular stress in AD, whereas AD-associated impairment of lysosomal function results in inadequate substrate clearance and blocked autophagy flux. For gradually declining substrate lysosomal clearance, sustained induction of autophagy clarifies the unusually strong autophagic pathology and neuritic dystrophy associated with the pathogenesis of AD [30]. These findings show that autophagy can regulate the renewal of soluble cytoplasmic proteins, decrease the accumulation of abnormal proteins, and prevent neurodegeneration.

### Autophagy in Neuronal Excitotoxicity

Neuronal damage caused by overactivation of excitatory amino acid receptors is called excitotoxicity, which is deemed to play a critical role in the pathogenesis of many neurological disorders, including AD, PD, and HD [31, 32]. Apoptosis is involved in excitotoxic neuronal death, as we and other laboratories have discovered in the early 1990s [33–35]. Excitatory amino acid receptor agonists were found to induce autophagy in animal models [36]. Autophagy was activated by N-methyl-D-aspartate (NMDA) receptor agonist quinolinic acid (QA) and kainic acid (KA) receptor agonist KA, which was then accompanied by decreased expression of Bcl-2, and increased expression of Bax, tumor suppressor gene that encodes for the transcription factor p53 (*TP53*), and *TP53*-upregulated modulator of apoptosis (PUMA). Autophagy activation and the mitochondria-mediated apoptotic pathway were markedly inhibited by autophagy inhibitors and cathepsin inhibitors, indicating that the autophagy-lysosomal pathway plays a significant part in excitotoxic neuronal injury [37, 38].

### Autophagy in Cerebral Ischemia and Ischemic Preconditioning

Globally, stroke is a major cause of death, more than 80% of which are ischemic stroke [39]. Preconditioning is usually a sub-threshold injury applied to an organ, activating specific endogenous protective pathways to buffer the damage from subsequent severe ischemic attack. It has been found that ischemic preconditioning (IPC) can protect against ischemic stroke [40, 41]. Autophagy is activated after ischemic stroke or preconditioning, but whether autophagy contributes to cell death or survival remains controversial [42].

#### Controversy Over the Role of Autophagy in Stroke

According to many studies, cerebral ischemia induces autophagy and results in neuronal death [43, 44]. Our study suggests for the first time that the ischemia-induced autophagy-lysosome pathway leads to neuronal death [45]. Later, autophagic cell death has also been reported to exist in adult and neonatal focal cerebral ischemia mouse models and in *in vitro* OGD models involving neurons [46] and vascular endothelial cells [47]. Knockdown of *Atg5* or *Atg7* to inhibit autophagy in different types of cells prevents neuronal death *in vivo* and *in vitro* [46]. Similarly, thrombolysis with PLAT/tPA can increase serum-free IGF1 and mediate neuroprotection by modulating the PI3K-AKT-mTOR pathway to reduce deleterious autophagy after cerebral ischemia [48]. Neferine, the main alkaloid in lotus seeds, was also able to significantly protect against cerebral ischemia by attenuating harmful autophagy in pMCAO models [49]. Asialo-rhuEPO^P^, purified from transgenic plant leaves, inhibits excessive mitophagy and autophagy induced by ischemia/reperfusion to reduce neuronal apoptosis and facilitate neuronal survival [50].

However, some studies support autophagy may have a protective effect on cerebral ischemia. Autophagy activation may represent a protective mechanism during the early stages of cerebral ischemia. A significant increase in Beclin 1 was observed in neurons in the hippocampus and cortex shortly after neonatal hypoxia-ischemia (HI). Autophagy inhibitors induced neuronal necrosis, whereas Rapamycin reduced both apoptosis and necrosis by promoting autophagy [51]. Further research suggests that autophagy is triggered during cerebral ischemia and reperfusion but produces different effects. Autophagy exerts a deleterious effect in permanent ischemia, whereas mitophagy activated during cerebral ischemia-reperfusion may produce a protective effect on neurons. Ischemia-reperfusion brain injury is aggravated by the inhibition of autophagy during reperfusion [52]. Recent studies provide further evidence for the protective effect of promoting autophagy initiation or mitophagy and accelerating the fusion of autophagosomes and lysosomes in cerebral ischemia-reperfusion [53, 54], hypertensive stroke [55], and subarachnoid hemorrhage (SAH) [56].

#### Autophagy Contributes to Ischemic Tolerance Induced by Preconditioning

One of the most effective methods to prevent an ischemic stroke is IPC [57]. Numerous studies, including our own, have shown that IPC also activates autophagy, and inhibiting autophagy abolishes IPC's neuroprotective effects [58–60]. Autophagy has also been shown to be concerned with the neuroprotection induced by hyperbaric oxygen preconditioning [61, 62], isoflurane preconditioning [63], or sevoflurane preconditioning [64, 65]. Although both fatal cerebral ischemia and IPC activate autophagy, the degree of this activation varies. Autophagy is excessively activated during permanent cerebral ischemia, while IPC moderately activates autophagy, and this activation extends to the subsequent ischemic event [42, 58]. Endoplasmic reticulum stress (ER stress) might be involved in the contradictory effects of autophagy during ischemia and preconditioning [59, 66]. IPC can upregulate molecular chaperones and activate autophagy, thereby reducing excessive ER stress-dependent apoptosis during lethal ischemia. The ER chaperone GRP78 might be an important regulator meditating autophagy activation during preconditioning [67]. Studies on ER stress and autophagy provide a strong basis for the use of IPC in the treatment of cerebral ischemia [58].

Interestingly, known pharmacological activation of autophagy can trigger autophagy to produce a protective effect against stroke (pharmacological preconditioning), including polyphenolic antioxidant resveratrol [68], visfatin [69] or nicotinamide [55] that mediate NAD^+^ biosynthesis, AMPK activator metformin [70] and trehalose [71]. The ischemic tolerance produced by these agents can be eliminated by inhibition of autophagy.

### Additional Neural Physiological and Pathological Conditions of Autophagy

Maintaining physical fitness and muscle mass in mammals necessitates consistent endurance activity. In mature and elderly animals, regular long-term exercise increases autophagy activity [72]. Interestingly, exercise also mitigates age-related cognitive decline by preserving mitochondrial quality control in the aged hippocampus through the autophagy-lysosomal pathway [73]. Exercise rejuvenates mitochondria and reduces oxidative stress in the aged hippocampus, as evidenced by increased activation of autophagy/mitophagy and mitochondrial biogenesis in aged hippocampal neurons. Additionally, exercise can enhance lysosomal function by promoting TFEB nuclear translocation and upregulating transcription of TFEB regulatory genes [74]. Chloroquine, a lysosomal inhibitor, partially disrupted the protective effects of exercise on mitochondrial quality control, oxidative stress, autophagy/mitophagy, and cognitive function in aged rats. These findings collectively suggest that age-related cognitive decline may be slowed down by exercise training or pharmacological modulation of lysosomal degradation and mitochondrial quality control [75].

During Traumatic brain injury (TBI), autophagy and lysosomal proteases in neurons are activated. Inhibition of the autophagy-lysosomal axis to relieve TBI and promote functional recovery is a viable approach [76]. However, contrary evidence suggests that TBI impairs autophagy flux and impedes Nrf2 signaling, a main regulator of antioxidant response, resulting in excessive oxidative stress. Calcitriol, the active form of vitamin D, reduces TBI-induced oxidative damage by facilitating autophagy and activating Nrf2 signaling [77].

Proteins and organelles quality control is necessary for normal synaptic function, and loss of autophagy may affect neuronal development [78, 79]. It has been proposed that synaptic activity can modulate the synaptic proteome by locally controlling autophagic vacuole (AVs) dynamics and function within dendrites. Stimulating synaptic activity inhibits AV movement in dendrites, thereby enhancing their degradability, whereas silencing synaptic activity has the opposite effect on AV function. This effect is localized and reversible and occurs in dendrites rather than axons, with compartmental specificity [80].

## Function of Autophagy in Neurological Disorders

### Autophagy Maintains Cell Homeostasis in the Nervous System

Neurons are terminally differentiated cells, and most of their synapses remain unchanged throughout the life cycle. During aging, the proteostasis network in neurons is disrupted and protein quality control is impaired, resulting in neurodegeneration. Neurons rely on autophagic machinery to clear damaged organelles and proteins to sustain synaptic neurotransmission and prevent neurodegeneration. Autophagy in neurons includes constitutive and stress-induced autophagy, which are involved in the renewal of damaged or aging endoplasmic reticulum, mitochondria, other organelles, and aggregate proteins [81, 82]. Under physiological conditions, neuronal soma contains populations of autophagosomes from different compartments with different mature states. Autophagosomes produced by axons enter the soma and are restricted to the somatodendritic domain, facilitating fusion with lysosomes within soma to promote cargo degradation. The autophagosomes produced in the soma are less mobile and tend to aggregate [83]. Moreover, in healthy neurons, autophagy regulates axonal endoplasmic reticulum calcium storage to modulate neurotransmission in the brain [84]. Protein aggregates disrupt neuronal homeostasis, leading to toxicity associated with neurodegeneration. Endogenous TAX1BP1 is an autophagy receptor protein that mediates the removal of a number of cytotoxic proteins. Overexpression of TAX1BP1 can facilitate autophagy and accelerate the removal of neuronal protein aggregates [85]. CMA plays a critical part in neuronal proteostasis, as CMA selectively degrades neurodegeneration-related proteins. Loss of neuronal CMA results in a senescent phenotype including reduced neuronal function, changes in neuronal metastable proteomes, and proteotoxicity, aggravating neuronal damage and accelerating disease progression in AD mice. Conversely, enhancement of CMA improves pathology in AD mice. Therefore, functional CMA is important for the maintenance of neuronal proteostasis and the reduction of misfolded proteins [86].

Adult neurogenesis is the process by which neural progenitor cells (NPCs) in the human brain continuously produce new functional neurons. Nevertheless, adult neurogenesis decreases with age, which is linked to neurodegeneration. The expression of autophagy-related genes and autophagy activity was significantly decreased in the cultured NPCs and middle-adult subventricular/subgranular zone (SVZ/SGZ) homogenates. In addition to restoring the vitality of middle-aged NPCs, activation of autophagy also stimulated neurogenesis in middle-aged SVZ, and improved neurological function and cognitive abilities in middle-aged animals. Conversely, the knockdown of autophagy-associated genes led to impaired NPC proliferation and differentiation. Thus, impaired autophagy is related to a decline in adult neurogenesis, while activation of autophagy can reverse this phenotype [87].

### Misfolded Protein Metabolism Involves Autophagy.

A variety of neurodegenerative disorders are characterized by the accumulation and aggregation of misfolded proteins, such as α-synuclein in PD, Huntington protein (HTT) in HD, and Aβ and tau protein in AD. Autophagy and the ubiquitin-proteasome system (UPS) represent the two primary mechanisms for degrading misfolded proteins that accumulate under pathological conditions. In various neurodegenerative diseases and cerebral ischemia, when the UPS is overloaded or damaged, the nervous system relies on autophagy to clear excessive misfolded proteins [88, 89].

The abnormal expansion of the polyglutamine (polyQ) tract in HTT protein causes HD [90]. Cortical pyramidal neurons and striatal projection neurons specifically degenerate in HD brains due to the aggregation of the N-terminal mutant HTT and the development of intranuclear inclusion [91, 92]. Many studies, including our own, indicate that lysosomal cathepsins and autophagy play essential parts in the degradation of N-terminal HTT [93–95]. Enhanced autophagy/lysosomal activity can significantly facilitate the degradation of mHTT and prevent the accumulation of mHTT aggregates [96]. Additionally, we also fully elucidated for the first time how macroautophagy [97] and CMA [98] degrade HTT fragments. Lysosome-associated protein 2A (LAMP2A) and heat shock protein cognate 70 (Hsc70) are CMA elements that are crucial for the clearance of HTT [98]. HTT is able to block the degradation of Beclin 1 and thus achieve autophagy. This is related to the polyQ domain in HTT-mediated interaction of the deubiquitinase ataxin 3 with Beclin 1. The longer polyQ mutations in the HTT protein competitively eliminate this function and inhibit autophagy [99]. K506 binding protein 5 (FKBP5) was markedly decreased in HD R6/2 and zQ175 rodent models. Genetic knockdown or pharmacological inhibition of FKBP5 can reduce the interaction between FKBP5 and HTT, resulting in mHTT being cleared by autophagy, accompanied by increased LC3-II and autophagy flux. The above suggests that the autophagic mechanism regulated by FKBP5 contributes to the neuronal clearance of mHTT [100]. In the adult brain, the specific and selective turnover of aggregated proteins is closely related to autophagy-linked FYVE protein (Alfy)/Wdfy3. In the HD mouse model, Alfy depletion and reduced autophagy level accelerate aggregated mutant huntingtin accumulation and promote behavioral deficits [101]. The miR-302 clusters were significantly downregulated in neuronal cells overexpressing mHTT-Q74. By restoring insulin sensitivity and autophagy, miR-302 reduces cytotoxicity induced by mutant huntingtin [102]. Phosphorylation of HTT proteins at S13 and S16 is essential for controlling their toxicity, aggregation, and removal. TBK1 overexpression increases mutant HTT's S13 phosphorylation, which prevents it from aggregating and encourages autophagic clearance of HTT aggregates [103]. The above results demonstrate that impaired autophagy contributes to the progression of HD, and enhancing autophagy to clear HTT aggregates may provide an efficient therapeutic strategy for HD.

The main pathogenic characteristics of AD are hyperphosphorylated tau protein and deposition of Aβ generated from amyloid precursor protein (APP). Blockade of autophagic clearance and lysosomal proteolysis have a close relation with neurodegeneration of AD [104–106]. In the AD models, the progression of macroautophagy requires the participation of the AD-associated protein presenilin-1 (PS1). Autolysosome acidification and cathepsin activation are impaired in PS1-depleted blastocysts. Impaired lysosomal clearance may be responsible for the PS1 deletion-mediated increase in Aβ [107]. In addition, there is an accumulation of large numbers of lysosomal-like organelles at amyloid plaques in AD mice, most of which are located within swollen axons in contact with amyloid deposits. Lysosomal precursors' retrograde axonal transport is hampered by extracellular Aβ deposits, resulting in their accumulation and preventing them from further maturing [108]. TFEB is an important lysosomal pathway regulator. In elderly mice, amyloid plaque load and total Aβ levels were reduced in the hippocampus transfected with TFEB. TFEB stimulates lysosomal biosynthesis, reduces steady-state levels of APP and α- and β-CTF, and attenuates Aβ production by accelerating the endosome-lysosomal pathway [109]. Microglia were found to be able to clear abnormally aggregated Aβ proteins through LC3-associated endocytosis [110] and autophagy [111]. However, in the microglia of adult AD mice, Aβ cannot be degraded and the expression level of the autophagy cargo receptor NBR is low. Inhibition of elevated miR-17 in microglia of AD mice increases NBR1, which in turn promotes autophagy and Aβ degradation [112]. In AD mice, miR-9-5p and miR-331-3p are downregulated in the initial stage, whereas upregulated in an advanced stage. Inhibition of miR-9-5p and miR-331-3p promotes autophagy degradation of Aβ and prevents AD progression [113]. Therefore, a sensible approach to treating neurotoxic Aβ may involve the upregulation of autophagy. Rapamycin [114], resveratrol [115], oxyresveratrol (OxyR) [116], and crocetin [117] can promote the degradation of Aβ, reduce neuroinflammation, and improve memory function by inducing autophagy, and thus may have therapeutic effects on AD mice. Similarly, PPARA/PPARα (peroxisome proliferation activated receptor α) agonists gemfibrozil and Wy14643 can promote the recruitment of astrocytes and microglia near Aβ plaques, and then induce autophagy in glia. Hence, in APP-PSEN1E9 mice, gemfibrozil and Wy14643 reduced soluble and insoluble Aβ levels alleviated amyloid pathology, and reversed anxiety symptoms and memory deficits [118].

The autophagy-lysosome pathway may also be essential for tau degradation. Most neuronal tau may be degraded by CMA. CMA dysfunction in the brains of patients with tauopathies alters tau protein homeostasis and may exacerbate disease progression. Acetylated tau inhibits CMA and leads to its extracellular release. In the mouse model of tauopathy, blocking CMA accelerated the spread of pathogenic tau proteins among cells [119]. The miR-9 target gene UBE4B, together with STUB1, enhances autophagy-mediated degradation of tau in the mouse model of Tau-BiFC, which may become an innovative AD treatment [120]. In a tau transgenic mice model, increased lysine acetyltransferase (p300/CBP) activity is connected with abnormal accumulation of autophagy lysosomal pathway markers. In neurons, overactivation of p300/CBP blocks autophagy flux and promotes tau secretion. On the contrary, in fibril-induced tau spreading models *in vivo* and *in vitro*, suppression of p300/CBP increased autophagic flux, and decreased tau secretion and spread, thereby blocking the progression of tauopathy disease in fibril-induced tau spreading models *in vivo* and *in vitro* [121]. These investigations showed that enhanced autophagy makes it easier to clear the aggregated proteins as Aβ and tau, thus preventing AD progression.

The pathological characteristic of PD is neuronal inclusions termed Lewy bodies, which consist of the misfolded protein α-synuclein. Autophagy plays a critical part in the clearance of α-synuclein [122]. Impaired autophagic flux in PD leads to lysosomal dysfunction and aggregation of α-synuclein proteins within dopamine neurons [123]. This results from α-Syn-mediated disruption of autophagosome-lysosome fusion, leading to reduced formation of autophagolysosomes. Mechanically, protein v-SNARE SNAP29 is a component of the SNARE complex, which facilitates the fusion of autophagosomes. By blocking SNAP29-mediated autophagosome-lysosome fusion, α-Syn inhibits autophagy and exacerbates the pathological damage in PD [124]. In addition, PD-like neurodegeneration caused by excess α-synuclein is also accompanied by a decrease in lysosomal function. Mitochondrial-derived reactive oxygen species (ROS) trigger abnormal permeabilization of lysosomal membranes, resulting in defects in lysosomal clearance, and ectopic release of lysosomal proteases into the cytoplasm contributes to neurodegeneration [125]. The PD pathology is also accompanied by cytoplasmic retention of TFEB, the main transcription factor of the autophagy-lysosomal pathway. Overexpression of TFEB can reverse lysosomal dysfunction in the brains of PD patients, thereby facilitating α-synuclein degradation for neuroprotective effects. Suppression of the mammalian target of rapamycin (mTOR) encourages TFEB nuclear translocation and blocks α-syn-induced neurodegeneration [126]. In addition, the CMA pathway can be used to specifically transport wild-type α-syn into lysosomes for degradation [127]. LAMP2A, a CMA receptor, is essential for the degradation of α-synuclein. Endosome-to-Golgi retrieval of LAMP2A is impaired in VPS35-deficient dopaminergic neurons, which affects the autophagic degradation of α-synuclein and accelerates the progression of PD [128]. Interestingly, microglia play an important role in clearing α-synuclein released by neurons. Microglia are activated by neuronal α-synuclein and subsequently engulf α-synuclein into autophagosomes and degrade them by selective autophagy called synucleinphagy. Synucleinphagy relies on the microglia Toll-like receptor 4 (TLR4). TLR4 improves the transcription of p62/SQSTM1 via the NF-κB signaling, mediating synucleinphagy to remove α-synuclein and produce neuroprotective effects [129]. Impairment of synucleinphagy leads to the accumulation of misfolded α-synuclein and loss of dopaminergic neurons [130]. Based on the above studies, enhancement of autophagy to promote the removal of α-synuclein may become an effective treatment for PD. Sestrin2 activates autophagy via AMPK and enhances the degradation of α-Synuclein, thereby protecting neurons from rotenone-induced dopaminergic neuronal damage [131]. Telomerase reverse transcriptase (TERT) reduces α-synuclein by activating autophagy or preventing disruption of the degradation machinery during disease progression [132]. C-Abl, a tyrosine kinase, is activated by cellular stress. In a TgA53T mouse model (human mutant A53Tα-Syn overexpressing transgenic mice), nilotinib inhibits c-Abl activity, increases autophagy flux via AMP-activated kinase (AMPK)/mTORC1/ULK1 signaling in neurons, thereby reducing the accumulation of α-synuclein and delaying the onset of the disease [133]. LRRK2 (leucine-rich repetitive kinase 2) mutations are thought to be a common cause of familial sporadic PD. Mutant LRRK2 disrupts CMA and impairs α-synuclein degradation [134]. Aged LRRK2 mutant striatum exhibits lysosomal aggregation, accumulation of LAMP2A and HSC70, and elevated GAPDH (CMA substrate). In mouse embryonic fibroblasts, the CMA-specific activator AR7 stimulates the transcription of LAMP2A and increases the activity of lysosomal. Besides, AR7 attenuates α-synuclein accumulation in cortical neurons with the LRRK2 mutation (DIV21) [135]. Therefore, enhancement of autophagy pathways to decrease the accumulation of pathogenic α-synuclein linked to aging may alleviate the progression of PD disease.

In the brains of patients with frontotemporal dementia (FTD) and ALS, brain inclusions consisting of misfolded and aggregated TDP-43 are a common pathological hallmark of ALS-FTD. Amplification of GGGGCC repeats in the *C9orf72* gene is the most frequent genetic cause of ALS-FTD [136], and polyglutamine-amplified Ataxin-2 (Ataxin-2 Q30x) is a genetic modifier of the ALS-FTD. C90ORF72 promotes TDP-43 clearance by enhancing autophagy, while depletion of C90ORF72 damages autophagy and leads to the accumulation of TDP-43 and p62 protein aggregates. Depletion of C90ORF72 works synergistically with Ataxin-2 Q30x to trigger motor neuron dysfunction and neuronal cell death [137]. Nuclear depletion of the *TARDBP* gene (encoding TDP-43) leads to neurodegeneration. Motor neurons lacking TARDBP/TBPH (the Drosophila homolog of *TARDBP*) in mouse/Drosophila models exhibit motor deficits and age-dependent neurodegeneration, reduced ATG7, and accumulation of SQSTM1/p62 inclusions. In TBPH-deficient flies, enhancement of autophagy improves motor function and survival [138]. In patient and cell models of ALS-FTD, TARDBP is cleaved to release a 25 kDa neurotoxic fragment (TARDBP-25/TDP-25) [139], and trehalose is able to induce removal of the aggregated TARDBP-25. Mechanistically, trehalose may trigger lysosomal membrane permeabilization (LMP) through lysosomal osmotic stress to release lysosomal Ca^2+^, thereby activating calcineurin and inducing TFEB dephosphorylation, which in turn clears misfolded proteins by enhanced autophagy [140]. In ALS, endogenous TDP-43 aggregates lose splicing regulation function. Thioridazine clears TDP-43 aggregates and restores TDP-43 function, thereby significantly ameliorating motion deficits in the ALS model [20]. Thus, the autophagy pathway can be used as a target for ALS-FTD and other related diseases presenting TARDBP pathology.

### Autophagy in the Elimination of Damaged Organelles

Elimination of damaged organelles and misfolded proteins depends on autophagy. Since neurons after mitosis are unable to reduce the level of misfolded proteins and damaged organelles by cell division, neurons highly depend on autophagy compared to other cells. Based on the selectivity of the engulfed component, autophagy can be divided into selective autophagy and non-selective autophagy. Selective autophagy is primarily responsible for the degradation of damaged organelles [141], including mitophagy, ER-phagy, or pexophagy.

**Fig. 2:**
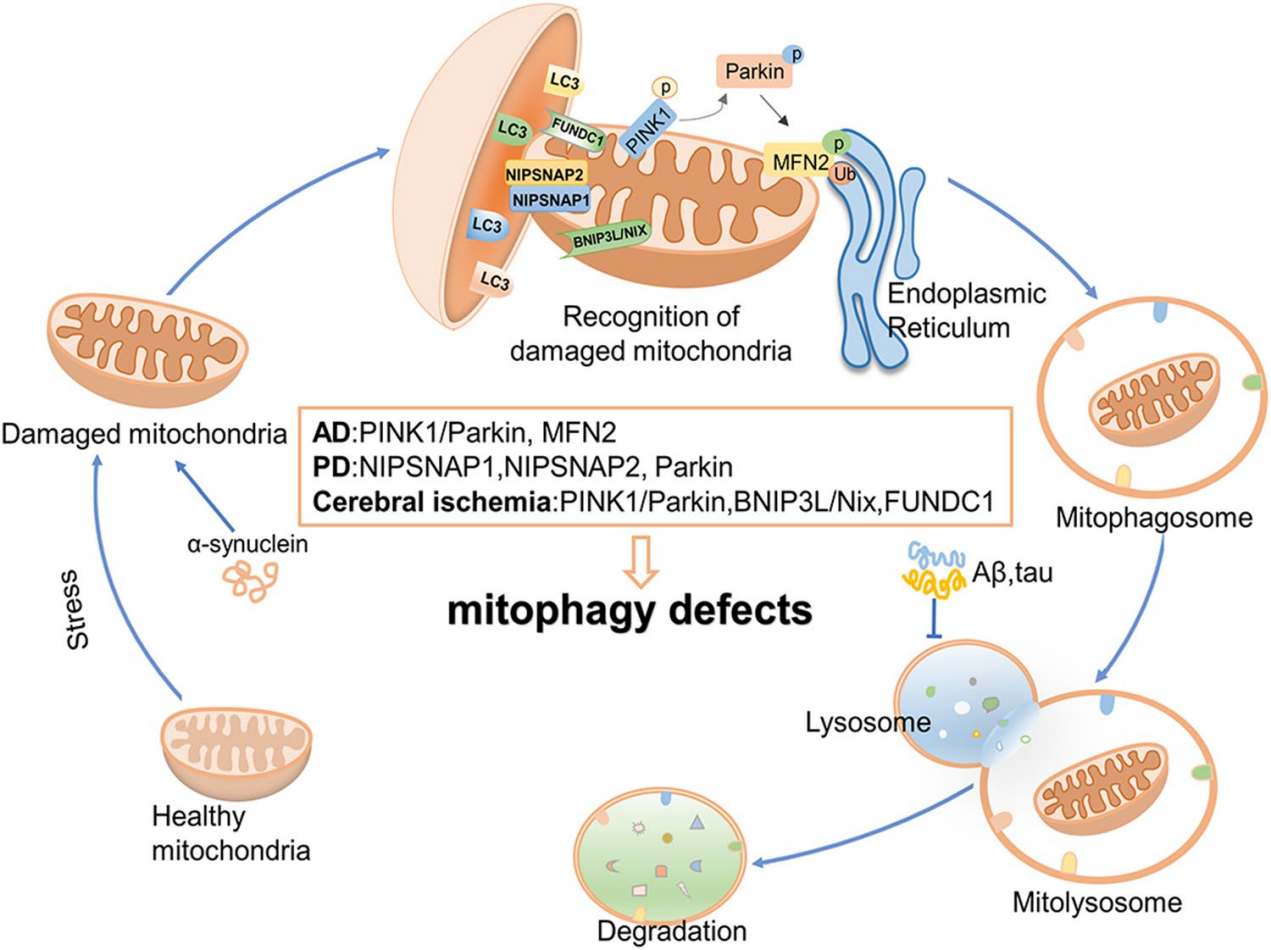
The role of mitophagy in neurodegenerative diseases and cerebral ischemia. Mitochondrial function is compromised during periods of stress. The process of mitophagy involves the identification of damaged mitochondria by double-membrane structures within the cytoplasm, followed by their encapsulation as mitophagosomes and subsequent degradation upon fusion with lysosomes. PINK1 and Parkin are two proteins located in mitochondria that play a crucial role in facilitating mitophagy. Specifically, PINK1 rapidly accumulates on damaged mitochondria and subsequently recruits Parkin to these organelles to promote phagocytosis. MFN2 is a mitochondrial-ER tethered protein. The ubiquitination of MFN2 by parkin, coupled with its phosphorylation by PINK1, triggers the disassembly of MFN2 complexes from the outer mitochondrial membrane. This process dissociates mitochondria from the ER and promotes their self-regulation. Inactivation of parkin can lead to defects in mitophagy, and accumulation of damaged mitochondria, and contribute to the development of PD and AD. BNIP3L/Nix and FUNDC1 are mitochondrial outer membrane proteins containing the LC3-interacting region (LIR) that mediate hypoxia-induced mitophagy. Amyloid β-protein (Aβ) and tau aggregation are two signature pathological hallmarks in AD. Mitophagy induction reduces Aβinhibits several common p-tau sites and improves memory impairment in AD mice. α-synuclein is involved in the development of PD. α-synuclein accumulation impairs the induction of mitophagy, increases the neuronal susceptibility to stress, and leads to dopaminergic neurodegeneration. NIPSNAP1 and NIPSNAP2, Mitochondrial matrix proteins, "autophagy" signals for damaged mitochondria; MFN2, mitofusin-2; FUNDC1, FUN14 domain-containing protein 1

Accumulation of damaged mitochondria tends to be present in aging and age-related neurodegeneration, including certain types of PD [142] and AD [143, 144] among others. Mitophagy is essential for the selective removal of damaged mitochondria to preserve mitochondrial homeostasis, ATP generation, and neuronal survival [145]. α-synuclein is related to the development of PD. Interestingly, the accumulation of α-synuclein impairs the induction of mitophagy and increases neuronal susceptibility to stress, leading to dopaminergic neurodegeneration and motor dysfunction [146]. The mitochondrial matrix proteins NIPSNAP1 (nipsnap homolog 1) and NIPSNAP2 (nipsnap homolog 2) are identified as "autophagy" signals for impaired mitochondria (Fig. 1). Following mitochondrial depolarization, NIPSNAP1 and NIPSNAP2 accumulate on the mitochondrial outer membrane and then promote autophagy by recruiting human Atg8 family proteins, other autophagy receptors and adapters. Zebrafish deficient in Nipsnap1 exhibit decreased mitophagy, increased ROS generation, dopaminergic neuronal loss in the brain, and decreased motility [147]. Similarly, in AD, mitochondrial disruption and metabolic dysfunction are early features before histopathological and clinical features, and mitochondrial dysfunction is also associated with synaptic defects in early AD. Enhanced lysosomal function in AD neurons promotes the clearance of damaged mitochondria, protects AD mouse brains from synaptic damage and restores impaired metabolic function [148]. Induced pluripotent stem cell-derived human AD neurons and the hippocampus of AD patients both exhibit impaired mitophagy. Aβ and phosphorylated tau inhibit mitophagy (Fig. 2). Mechanistically, mitophagy reduces insoluble Aβ1-40 and Aβ1-42 and prevents cognitive disorder in the APP/PS1 mouse model via suppression of neuroinflammation and microglial phagocytosis of extracellular Aβ plaques. Besides, enhanced mitophagy abolished AD-associated tau hyperphosphorylation in neurons and improved memory deficits in mice and transgenic tau nematodes [18].

Mutations in two PD-related genes, *PARK*2 (encoding Parkin, an E3 ubiquitin ligase) and *PARK*6(encoding PINK1, PTEN-induced kinase 1, a ubiquitin kinase) can lead to early-onset PD. Patients with PD are usually accompanied by mitochondrial dysfunction [149, 150]. Parkin and PINK1 selectively identify damaged or depolarized mitochondria to mediate mitophagy and prevent an increase in cytosolic and circulating mitochondrial DNA, thereby reducing neuroinflammation and neurodegeneration in PD [151, 152]. PINK1 expression on individual mitochondria is regulated by voltage-dependent proteolysis, which is important for maintaining low levels of PINK1 in normal mitochondria. However, PINK1 rapidly accumulates on damaged mitochondria and recruits Parkin to mitochondria. PINK1 and Parkin then induce mitochondrial ubiquitination, resulting in phagocytosis of damaged mitochondria [153]. Loss of Parkin activity causes the dysfunctional mitochondria to accumulate [154], resulting in the loss of neurons in PD [155]. In AD, parkin and PINK1 can also mediate mitophagy to selectively remove damaged mitochondria [154]. MFN2, a mitochondrial-ER tethering protein, regulates mitophagy through PINK1 and Parkin. Parkin-mediated MFN2 ubiquitination coupled with PINK1-catalyzed MFN2 phosphorylation triggers the disassembly of the P97-dependent MFN2 complex from the mitochondrial outer membrane, thereby disconnecting mitochondria from ER and facilitating mitophagy (Fig. 2) [156]. Therefore, PINK1/Parkin plays a critical part in regulating mitophagy and mitochondrial quality control [157]. Mitophagy inducers (NAD^+^, actinonin, and urolithin A) restore memory impairment through the PINK1/Parkin-dependent pathway [18, 158]. Consistently, in a motoneuronal death model, GRP78 overexpression promotes PINK1 translocation to induce mitophagy and restores mitochondrial function in cells upon ER stress [159]. Thus, the impaired clearance of dysfunctional mitochondria is a major contributor to the pathogenesis of neurodegenerative disorders, and mitophagy is considered as a viable therapeutic strategy.

Mitophagy is also able to alleviate cerebral ischemia-induced neuronal injury by selectively eliminating dysfunctional mitochondria [160]. Interestingly, except for the PINK1/Parkin pathway, mitophagy may not be dependent on the PINK1 pathway in some tissues with high metabolic demand [161]. The mitophagy receptors BNIP3L/Nix [162] and FUNDC1 [163] are both mitochondrial outer membrane proteins containing the LC3 interaction region (LIR) that have been shown to mediate hypoxia-induced mitophagy and play important roles in mitophagy-induced by cerebral ischemia (Fig. 2). It has been found that mitophagy activated by cerebral ischemia-reperfusion may rely on the PINK1/Parkin pathway at the early stage. Parkin-mediated mitophagy induced by acidic postconditioning (APC) [164] or hypoxia postconditioning (HPC) [165] prolongs the reperfusion time window and exerts neuroprotective effects in stroke. However, cerebral ischemia-induced mitophagy at the later stage may depend on the BNIP3L/Nix pathway. BNIP3L deficiency inhibited mitophagy and exacerbated cerebral ischemic injury in mice, while overexpression of BNIP3L can reverse these effects. Phosphorylation of BNIP3L S81A plays a critical role in BNIP3L-mediated mitophagy [166]. BNIP3L/NIX can be degraded by proteasomes, resulting in defective mitophagy in ischemic neurons. The proteasome inhibitor carfilzomib reverses BNIP3L degradation and restores mitophagy, thus preventing ischemic brain injury [167]. Interestingly, in neurons exposed to ischemia-reperfusion, axonal mitochondria are retrogradely transported into neuronal soma and preferentially eliminated by mitophagy. Anchoring of syntaphilin to axonal mitochondria blocks neuronal mitophagy and aggravates damage. Conversely, induction of mitochondrial retrograde transport enhances mitophagy, prevents mitochondrial dysfunction, and alleviates neuronal damage [168]. During an acute ischemic stroke, PPAR coactivator-1α (PGC-1α), the major regulator of mitochondrial biogenesis, is rapidly elevated in microglia. PGC-1α facilitates mitophagy and autophagy through ULK1, thereby inhibiting NLRP3 activation and attenuating neurological deficits after ischemic injury [169]. Tissue plasminogen activator (tPA) promotes mitophagy by regulating FUNDC1. tPA thus improves mitochondrial function and inhibits apoptosis in neurons to protect against ischemic reperfusion injury [170].

Selective autophagy degradation of endoplasmic reticulum (ER) fragments under normal or stress stimuli is termed ER-phagy or reticulophagy. ER-phagy is a crucial mechanism for preventing ER stress-related ER expansion and offers an alternative disposal pathway for misfolded proteins [171, 172]. Atg39 and Atg40 [173], two autophagy-related proteins identified in yeast, are located in the perinuclear ER and cytoplasmic ER to mediate nucleophagy and ER-phagy, respectively. The mammalian homolog of Atg40, FAM134B, belongs to the reticulon protein family. FAM134B was found to bind to LC3 and GABARAP and mediate ER-phagy. FAM134B deficiency results in impaired ER turnover, increased cell sensitivity to stress-induced apoptotic death, and degeneration of sensory neurons [174]. VPS13, a conserved phospholipid transporter, is found at the contact sites between ER and different organelles. VPS13A is located at the ER-mitochondrial contact site, while VPS13C is located at the ER-late endosome contact site. Loss of VPS13C function increases the vulnerability of mitochondrial to stress and triggers excessive mitophagy, which may be a key issue in autosomal-recessive early-onset parkinsonism [175]. Interestingly, the impaired ER-phagy triggered by VPS13 deletion may further explain the importance of VPS13C in early-onset Parkinson’s. When VPS13 is absent, ER accumulation in late endosomes and ER transport into autophagosomes are decreased, suggesting a role for VPS13 in sequestering ER from late endosomes to autophagosomes [176]. Thus, ER-phagy is essential for maintaining the homeostasis of mammalian cells, and its dysfunction may be closely linked to the development of neurodegenerative disorders.

### Autophagy in Regulation of Apoptosis

Autophagy plays a crucial role in maintaining cellular homeostasis, degrading misfolded proteins, and eliminating damaged organelles, thereby promoting cell survival under stress conditions. However, the role of autophagy in cell survival and death is highly intricate. While autophagy determines the fate of organelles, apoptosis decides the destiny of entire cells. Autophagy can induce "type II programmed cell death" by bulk degradation of intracellular organelles and cytosol, which distinguishes it from apoptosis (type I programmed cell death). Studies have demonstrated that autophagy and apoptosis coexist in neurological diseases, with autophagy potentially promoting apoptosis. Bcl-2 is a known anti-apoptotic protein that has been found to interact with the autophagy protein Beclin 1, resulting in the inhibition of autophagy. Mutations in Beclin 1 that prevent binding to Bcl-2 induce stronger autophagy and promote cell death compared to wild-type Beclin 1 [177]. Serum deprivation-induced autophagy activation was accompanied by upregulation of Bcl-2. Downregulation of Bcl-2 expression or pharmacological inhibition of Bcl-2 function enhanced autophagy activation and led to apoptosis, while overexpression of Bcl-2 hindered autophagy activation and inhibited cell death caused by serum deprivation [178]. These studies have provided important information on the classical role of autophagy on apoptosis in nutritional stress. In KA and NMDA receptor agonist QA-induced apoptosis, the autophagy inhibitor 3-methyladenine (3-MA) blocked the down-regulation of pro-survival protein Bcl-2, thus inhibiting apoptosis [37]. The neuronal death induced by mitochondrial inhibitor 3-nitro propionic acid (3-NP) involves both apoptosis and autophagy. 3-NP upregulates *TP53* and its downstream genes *BAX*, *PUMA,* and *DRAM1*. Noticeably, neuronal damage caused by 3-NP was inhibited by the autophagy inhibitor 3-MA, the *TP53* specific inhibitor pifithrin-α, and by knock-down of DRAM1, suggesting that *TP53* and DRAM1 play important roles in 3-NP-induced autophagy and apoptosis [38]. DRAM1 regulates autophagy by enhancing the acidification of lysosomes, fusion of autophagosomes with lysosomes, and removal of autophagosomes [179]. DRAM1 also regulates the crosstalk between autophagy and apoptosis by interacting with BAX. DRAM1-mediated activation of autophagy upregulates BAX and recruits it into lysosomes. BAX initiates apoptosis via lysosomal protease cathepsin B-mediated Bid cleavage, cytochrome c release, and caspase 3 activation. These findings imply that DRAM1 regulates apoptosis and autophagy in cells by inhibiting BAX degradation [180].

However, contrary evidence points to the inhibition of apoptosis caused by the activation of autophagy, which may be connected to the removal of damaged mitochondria. Rapamycin facilitates translocation of p62 and parkin to impaired mitochondria to activate mitophagy and reduces cytochrome c release by inhibiting spinal cord ischemia-reperfusion injury (SCIRI)-induced Bax translocation to mitochondria, thereby inhibiting apoptosis and protecting neurons [181]. Necroptosis, a programmed necrosis, is activated in AD and may be associated with impaired autophagy. UVRAG is downregulated in AD, resulting in impaired autophagy flux and p62 accumulation. Accumulated p62 then recruits RIPK1 and triggers its self-oligomerization, which ultimately leads to neuronal necroptosis through the RIPK1/RIPK3/MLKL cascade [182]. Thus, the complicated relationship between autophagy and apoptosis may be attributed to different regulatory signals in different disease processes.

### Autophagy and Neuroinflammation

In neurodegenerative diseases like AD and PD, neuroinflammation plays a significant role [183, 184]. Anti-inflammatory therapy thus has been recognized as a viable therapeutic approach. Autophagy may affect neurodegenerative diseases by regulating microglia-mediated neuroinflammation. Mice with structural abnormalities of the autophagy protein Atg16L exhibited reactive microgliosis, Aβ deposition, tau hyperphosphorylation, pervasive neurodegeneration, and severe memory and behavioral deficits. Pharmacological inhibition of neuroinflammation alleviated memory impairment and pathology in this AD model [185]. In AD, toxic Aβ oligomers (AβOs) impair autophagy in microglia and induce neuroinflammation. Achyranthes bidentate polypeptide fraction k (ABPPk) can restore autophagy in AβOs-injured microglia to restrain the M1 phenotype and facilitate the M2 phenotype, leading to anti-inflammatory activity. ABPPk thus significantly improves locomotor activity and alleviates memory deficits in AD [186].

In addition, the loss of microglia Atg5 leads to PD-like symptoms in mice, manifested as impaired cognitive learning and motor coordination, tyrosine hydroxylase (TH) neuronal loss, enhanced neuroinflammation, and decreased dopamine in the striatum. When autophagy was suppressed, NLRP3 inflammasome in microglia was activated, accompanied by sustained upregulation of downstream IL-1β and increased expression of the pro-inflammatory cytokine MIF (macrophage migration inhibitory factor)**.** NLRP3-specific inhibitor MCC950 can prevent the formation of NLRP3 inflammasome, reduce MIF expression and neuroinflammatory response, and rescue TH neuronal loss in the substantial nigra (SN) [187]. In type 2 diabetic mice, metabolic inflammation accelerates the degradation of dopaminergic neurons. Metformin inhibits neuroinflammation induced by hyperactivation of microglia in the substania nigra compacta in mice with PD, thereby alleviating the degeneration of dopaminergic neurons [188].

Neuroinflammation and neurodegeneration are the main causes of progressive disability in patients with Multiple sclerosis (MS). In the MS mouse model, the recovery of CNS inflammation depends on the ability of microglia to remove tissue fragments. Loss of Atg7 in microglia results in the accumulation of phagocytic myelin and progressive MS-like lesions. Therefore, inducing autophagy in elderly mice promotes the clearance of functional myelin and alleviates the disease. Trehalose enhances the autophagy of microglia and promotes the removal of tissue debris, thereby inhibiting inflammation and relieving MS [189].

According to the aforementioned findings, regulating autophagy may offer a potential new therapeutic strategy for neurodegenerative diseases associated with neuroinflammation.

## Signaling to Regulate Autophagy in Neurological Diseases

### AMPK- mTOR-dependent Pathway

mTOR, a serine/threonine protein kinase, regulates cell growth, reproduction, and protein synthesis by integrating intracellular and extracellular signals [190]. 5'-AMP-activated protein kinase (AMPK) is an upstream of mTOR and can sense the energy state of cells. In the case of energy stress or low energy, AMPK is activated, inhibits mTOR [191] then enhances autophagy. The key mediator of autophagy, ULK1-ATG13-ATG101-FIP200, is primarily regulated by AMPK-mTOR. Under normal circumstances, activated mTOR can phosphorylate the ser757 site of ULK1, interfering with the interaction between AMPK and ULK1, thus preventing AMPK from activating ULK1 and autophagy. Under cellular stress, activated AMPK can inhibit mTORC1 to alleviate ULK1 Ser 757 phosphorylation. Conversely, AMPK phosphorylates Ser777 and Ser 317 on ULK1 to activate ULK1 kinase, which ultimately induces autophagy [192, 193].

The AMPK-mTOR pathway has an important effect on the pathogenesis of many neurodegenerative diseases. Activation of mTOR mediates AD progression by inhibiting autophagy and reducing the clearance of Aβ [194]. Activating AMPK inhibits mTOR activity, enhances autophagy, and facilitates Aβ degradation [195]. However, researchers have found that mTOR signaling is indispensable for synaptic plasticity and memory formation in the hippocampus, and restraint of mTOR disrupts memory consolidation [196]. In APP/PS1 transgenic mice, transient receptor potential mucolipin-1 (TRPML1) was downregulated, accompanied by activation of AMPK signaling, and inhibition of mTOR signaling. Overexpression of TRPML1, or treatment with AMPK inhibitors and mTOR activators can downregulate ALR-associated proteins, improve recognition and memory impairment, and alleviate neuronal apoptosis, suggesting that TRPML1 participates in the pathogenesis of AD by modulating autophagy through AMPK-mTOR signaling [197].

The AMPK-mTOR pathway also mediates the activation of autophagy and produces a protective effect during ischemia or preconditioning. Metformin and resveratrol can activate AMPK, induce autophagy or mitophagy, and provide protection against subsequent cerebral ischemia [68, 70]. Metformin preconditioning and autophagy activation are hindered by the AMPK inhibitor Compound C or the autophagy inhibitor 3-MA. Rapamycin specifically inhibits mTORC1 to induce autophagy, which has been proved to be related to the protective effect of rapamycin on cerebral/myocardial ischemia [198, 199]. Hippocampal CA3 neurons are stimulated by ischemia to produce hamartin, which is a product of the tuberous sclerosis complex 1 gene (TSC1). Inhibition of the expression of hamartin increases the vulnerability of neurons to cell death after ischemia, whereas overexpression of hamartin protects neuronal against ischemia injury through mTORC1-dependent mechanism that induces autophagy [60]. These data suggest that agents acting on the AMPK-mTOR pathway to induce autophagy may have the potential to treat neurodegenerative diseases or ischemic cardiovascular diseases.

### Beclin 1 and autophagy

*Beclin 1*, a mammalian homolog of yeast Atg6, is an autophagy-related tumor suppressor gene [200]. Bcl-2 or Bcl-XL binds to the BH3 domain of Beclin 1 to inhibit Beclin 1. In addition, BH3-only protein or pharmacological BH3 mimics can trigger autophagy by competitively interfering with the interaction between Bcl-2 or Bcl-XL and Beclin 1 [201].

Beclin 1-mediated autophagy may mediate neuroprotection in brain preconditioning. By triggering autophagy via the HIF-1α/Beclin 1 pathway, hypoxic preconditioning (HPC) reduces OGD/R-induced damage to SH-SY5Y cells [202]. TOM7 is an important part of the protein translocase of the outer mitochondrial membrane (TOM) complex. TOM7 can modulate autophagy through the PINK1/Beclin 1 signaling after cerebral ischemia, and silencing TOM7 inhibits autophagy via the PINK1/Beclin 1 pathway to aggravate cerebral ischemia [203]. Sphingosine kinase 2 (SPK2) leads to autophagy activation induced by isoflurane or hypoxia preconditioning in neurons and participates in the neuroprotection of cerebral preconditioning [204]. Interestingly, SPK2-mediated autophagy and protection is independent of its catalytic activity but displaces Beclin 1 from the Bcl-2/Beclin 1 complex through its BH3 domain, thus releasing free Beclin 1 to induce autophagy [63, 205]. The Tat-SPK2 peptide designed according to the BH3 domain of SPK2 can protect neurons against ischemic injury by activating autophagy or mitophagy [206].

Beclin 1-mediated autophagy has also been linked to some neurodegenerative diseases. In PC12 cells, Beclin 1 may exert an effect on the autophagic degradation of the mutant HTT552. Blockade of Beclin 1 nuclear output or reduction of Beclin 1 expression induced the formation of mHTT552 aggregates [97]. In addition, Aβ can stimulate the production of inflammatory cytokines, thereby inhibiting the Beclin 1-mediated autophagy and the capacity of microglia to phagocytosis Aβ [207, 208]. Beclin 1-dependent autophagy can not only affect AD pathology by regulating the microglial phagocytosis of Aβ but may also affect the production and release of cytokines leading to progressive neuroinflammation [209].

### *TP53* and *TP53* Target Genes (*DRAM, TIGAR*) and Autophagy

*TP53* is a key tumor suppressor gene product that is essential in apoptosis, DNA repair, and cell cycle regulation. In addition to its important role in carcinogenesis, a growing amount of research has found that *TP53* and its target gene (*TIGAR, DRAM1*) play important roles in neurological diseases by regulating autophagy [210, 211].

*TP53* and *DRAM1* in particular may be critical factors mediating autophagic activation and mitochondrial dysfunction in neuronal excitotoxicity. Inhibition of *TP53* suppresses autophagy activation, mitochondrial dysfunction, and excitatory neuronal damage [212]. DRAM1 may affect the autophagy machinery at multiple levels. DRAM1 can enhance lysosomal acidification, and lysosomal fusion with autophagosomes, and promote autophagy flux. Knockdown of DRAM1 exerts an inhibitory effect on autophagy by disrupting autophagosome-lysosomal fusion, thereby exacerbating OGD/R-induced cell damage in neuro-2a [213]. DRAM1 also induces autophagy by inhibiting the PI3K-AKT-mTOR-S6k pathway [214]. DRAM1 is not only required for *TP53*-mediated apoptosis but also regulates the crosstalk between autophagy and apoptosis through interacting with BAX, as previously described [180].

*TP53*-induced glycolysis and apoptosis regulator (TIGAR) is capable of inhibiting glycolysis through fructose-2, 6-diphosphatase activity to facilitate the pentose phosphate pathway, increasing intracellular NADPH and decreasing ROS. TIGAR thus inhibits autophagy activation by inhibiting ROS [215, 216]. TIGAR shows neuroprotection in monkey and rodent models of cerebral ischemia. Mechanistically, TIGAR partially inhibits autophagy by decreasing ROS and activating the mTOR-S6K pathway, thereby preventing cerebral ischemia-induced neuronal injury [217]. Moreover, TIGAR defends the tight junction of brain microvascular endothelial cells by preserving NADPH production and preventing autophagy [218]. However, other findings suggest the role of TIGAR in inducing autophagy. In high-glucose-stimulated neuronal cells and the hippocampus of streptozotocin (STZ)-induced diabetic mice, overexpression of TIGAR rescued the impaired autophagy induced by high-glucose and reduced neuronal apoptosis [219]. Interestingly, TIGAR’s neuroprotection in long-term cerebral ischemia by alleviating oxidative stress was independent of the pentose phosphate pathway triggered by its phosphatase activity. In the brain with chronic ischemia, TIGAR induced autophagy, thereby activating Nrf2 and producing a sustained antioxidant effect [220]. The above results suggest that TIGAR can inhibit or induce autophagy through phosphatase activity or non-phosphatase activity under different pathological conditions.

### ER Stress and Autophagy

During ER stress, active transcription factor 6 (ATF6), inositol-dependent enzyme 1 (IRE1), and protein kinase R-like endoplasmic reticulum kinase (PERK) dissociate from GRP78 and are activated to initiate unfolded protein response (UPR). Subsequently, the expression of UPR-related genes including molecular chaperones is increased, while the overall protein translation levels are downregulated to restore ER homeostasis [221, 222]. ER stress at the early stage can prevent protein aggregation by inhibiting protein synthesis and upregulating ER chaperone protein expression (such as GRP78, GRP94, HSPs, etc.) to facilitate the proper folding of proteins, thus promoting the restoration of ER function and protecting cells. However, persistent ER stress may lead to the activation of CHOP and caspase-12, thus triggering ER stress-dependent apoptosis [223].

**Fig. 3:**
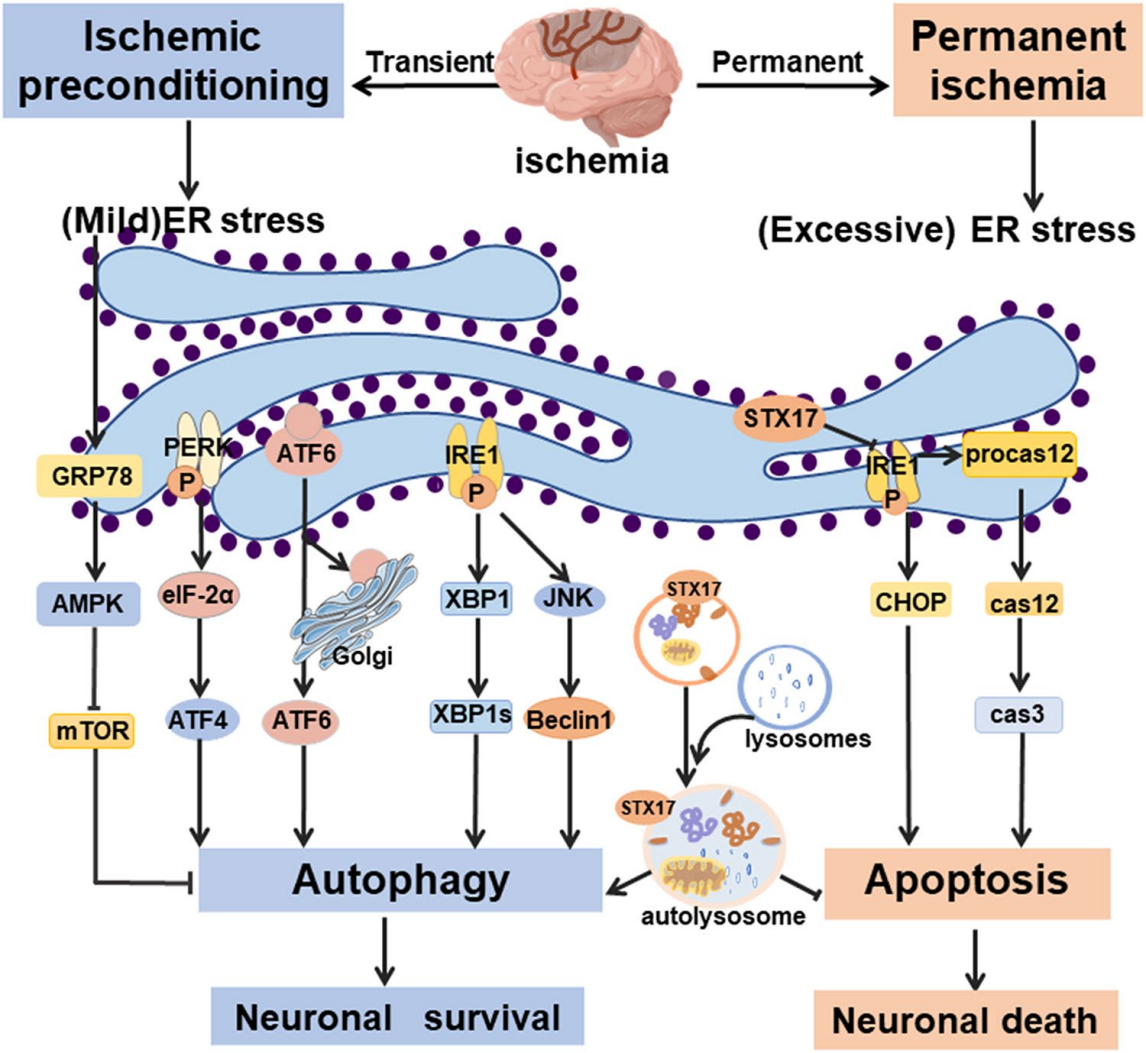
Endoplasmic reticulum stress and autophagy in cerebral ischemia and ischemic preconditioning. In the case of insufficient energy supply or nutrient deficiency, misfolded or unfolded proteins accumulate in the ER lumen, triggering ER stress through signals of unfolded protein-responsive receptors IRE1, PERK, and ATF6. Cerebral ischemic preconditioning induces mild ER stress and upregulates ER chaperone GRP78 to activate mild autophagy through the AMPK/mTOR pathway, producing a neuroprotective effect. However, permanent cerebral ischemia leads to severe ER stress, increased CHOP expression, cleavage of caspase 12, and apoptosis. Mild autophagy induced by STX17 also prevents ER stress-dependent apoptosis in fatal ischemia. GRP78, glucose-regulated protein of molecular mass 78; IRE1α, Inositol-requiring transmembrane kinase endoribonuclease-1α; ATF6, Activating Transcription Factor 6; XBP1, a master regulator of the unfolded protein response; JNK, c-Jun N-terminal kinase; STX17, Syntaxin 17; CHOP, C/EBP homologous protein; cas3, Caspase3; cas12, Caspase12

ER stress is associated with the differential effects of autophagy during ischemia and preconditioning [59, 66]. ER stress has been demonstrated to play a dual role in lethal ischemia and preconditioning [58, 59]. ER stress induced by lethal ischemia leads to activation of CHOP and caspase-12 [224, 225]. However, preconditioning may increase the levels of heat shock proteins (HSPs) or ER chaperones to alleviate the intense ER stress that occurs during lethal ischemia [226, 227]. Preactivation of autophagy by IPC can reduce excessive ER stress-dependent apoptosis during fatal ischemia, accompanied by upregulation of chaperones HSP60, HSP70, and GRP78 and downregulation of CHOP and caspase-12 [60]. Interestingly, the neuroprotective effect of IPC can be eliminated by the inhibition of endoplasmic reticulum stress, accompanied by autophagy inhibition [66]. Endoplasmic reticulum stress inducers tunicamycin (TM) or thapsigargin (TG) can also effectively alleviate fatal cerebral ischemia injury by inducing autophagy [228]. The ER chaperone GRP78 may be a critical mediator of autophagy activation in preconditioning [67]. Similarly, mild autophagy induced by STX17 prevents ER stress-dependent apoptosis in fatal ischemia [53]. In subarachnoid hemorrhage (SAH), the autophagy protein NRBF2 reduces neuroinflammation and oxidative stress associated with ER stress by interacting with Rab7 to promote autophagosome maturation [56]. These studies collectively provide good evidence for the idea that preconditioning can induce mild ER stress to upregulate molecular chaperones, contributing to autophagy activation, and thereby preventing ER stress-dependent apoptosis in fatal cerebral ischemia (Fig. 3).

In addition, PD is affected by ER stress-mediated autophagy. In response to ER stress, the transcription factor XBP1 is unconventionally spliced and activated by ERN1/IREα. XBP1 then activates PINK1 in neurons and triggers mitophagy that relies on endogenous PINK1. PINK1 kinase can control the transcriptional activity of XBP1s by its phosphorylation. The translocation of XBP1s to the nucleus is facilitated by PINK1-mediated phosphorylation, favoring its transcriptional activity and enhancing the transcription of PINK1 itself. However, the XBP1-PINK1 circuit that controls mitophagy may be disrupted in PD, leading to ER stress and mitochondrial dysfunction, thereby promoting the development of PD [229].

### FOXO and Autophagy

FOXO, a transcription factor, regulates a variety of cellular functions such as cell differentiation, metabolism, proliferation, and survival. Moreover, FOXO is involved in the regulation of autophagy [230]. FOXO1 may induce the expression of BNIP3, which then replaces Beclin 1 in the Beclin 1/Bcl-XL complex to initiate autophagy [231]. The ACE2/Ang(1-7)/MasR axis induces FOXO1 and autophagy flux and inhibits the transition of microglia polarization from M1 to M2 phenotype, thereby suppressing inflammation and mediating neuroprotection [232]. FKBP5 is upregulated in cerebral ischemia-reperfusion injury and is connected with the severity of neuronal injury. FKBP5 activates autophagy by preventing the phosphorylation of AKT and FOXO3 and exacerbates neuronal injury, while FKBP5 knockdown alleviates ischemic neuronal damage [233]. This suggests that FOXO1/3-mediated autophagy is involved in microglia-mediated neuroimmunomodulation and neuronal survival.

## Conclusion and Perspective

In general, autophagy plays diverse roles in neuronal excitotoxicity, neurodegenerative diseases, ischemic preconditioning, and cerebral ischemia. Mild autophagy eliminates damaged organelles and harmful protein aggregates from cells, thereby limiting the spread of detrimental signals. However, excessive autophagy leads to permanent organelles damage, triggering cell death. Autophagy is involved in maintaining cellular homeostasis by coordinating with other cellular activities, including mitochondrial function, endoplasmic reticulum stress, and apoptosis. The interplay of autophagy with different essential elements in various signaling pathways involving AMPK-mTOR, Beclin 1, *TP53*, and DRAM1 may explain its multifunctional role.

Although much research has been done on the role of autophagy in neurological diseases, there are still some issues that need to be addressed. First, mild to moderate autophagy may be a pro-survival mechanism for neurons to maintain CNS homeostasis. However, sustained or excessive autophagy may result in autophagic cell death. Therefore, how to activate moderate autophagy to exert the benefit effect of autophagy and prevent its harmful effects is a critical issue that needs to be addressed urgently in clinical application. Second, what is particularly interesting is the differential role of autophagy in ischemic stroke. Contradictory evidence has shown that activation or inhibition of autophagy may produce neuroprotection in ischemic stroke, which may be associated with the different stages in ischemia or reperfusion and different signaling that mediate autophagy. Therefore, investigating the differential role of autophagy in different stages of ischemia and its regulatory signals will enable precise regulation of autophagy to prevent neuronal injury in the future. Third, neurodegenerative diseases and stroke are more common in the elderly, and the research on the effect of autophagy on neurological diseases is mostly based on experiments on cells and young animals. Therefore, the existing research has the problem of large differences in age and species and there is still a long distance from clinical translations. In addition, for central nervous system drugs, how to penetrate the blood-brain barrier and target the nervous system is a key issue to improve drug efficacy and reduce side effects. Therefore, how to improve the value of autophagy in the clinical application of neurological diseases is a main direction for future research. A better understanding of autophagy and its regulatory mechanisms will provide useful information for insight into the pathogenesis of central nervous system diseases and the development of new therapeutic approaches.
